# Children's unique experience of depression: Using a developmental approach to predict variation in symptomatology

**DOI:** 10.1186/1753-2000-1-9

**Published:** 2007-08-22

**Authors:** Misty M Ginicola

**Affiliations:** 1 Yale University, 310 Prospect St. New Haven, CT, 06511, USA

## Abstract

**Background:**

Current clinical knowledge suggests that children can have different types of depressive symptoms (irritability and aggression), but presents no theoretical basis for these differences. Using a developmental approach, the present study sought to test the relationship between developmental level (mental age) and expression of depressive symptoms. The primary hypothesis was that as children's mental age increased, so would the number of internalizing symptoms present.

**Methods:**

Participants were 252 psychiatric inpatients aged 4 to 16 with a diagnosed depressive disorder. All children were diagnosed by trained clinicians using DSM criteria. Patients were predominantly male (61%) with varied ethnic backgrounds (Caucasian 54%; African American 22%; Hispanic 19%; Other 5%). Children were given an IQ test (KBIT or WISC) while within the hospital. Mental age was calculated by using the child's IQ score and chronological age. Four trained raters reviewed children's records for depressive symptoms as defined by the DSM-IV TR. Additionally, a ratio score was calculated to indicate the number of internalizing symptoms to total symptoms.

**Results:**

Mental age positively correlated (*r *= .51) with an internalizing total symptom ratio score and delineated between several individual symptoms. Mental age also predicted comorbidity with anxiety and conduct disorders. Children of a low mental age were more likely to be comorbid with conduct disorders, whereas children with a higher mental age presented more often with anxiety disorders. Gender was independently related to depressive symptoms, but minority status interacted with mental age.

**Conclusion:**

The results of this study indicate that a developmental approach is useful in understanding children's depressive symptoms and has implications for both diagnosis and treatment of depression. If children experience depression differently, it follows that treatment options may also differ from that which is effective in adults.

## Background

Depression can be found in a wide range of individuals, from infants to the elderly [1,2]. However, research indicates that children's experience of depression differs significantly from that evidenced in adults. The current perspective on depression, as indicated in the Diagnostic and Statistical Manual of Mental Disorders [1] and the National Institute for Clinical Excellence [3], suggests that, although children and adults can have similar symptoms, their presentation may vary. Past reports have indicated the presence of irritability and aggression as symptoms of depression in children, whereas these symptoms are not listed as evident in depressed adults [4].

One possible reason for these findings is that children normatively develop cognitive and emotional skills over the course of their childhood [5]. Prior to reaching some of these cognitive milestones, they normatively present with a more behavioral and less cognitive orientation to their environment. Developmental research indicates that externalizing behaviors are present at low developmental levels (young children) and gradually change to internalizing behaviors over time.

Based upon the conceptualizations of behavior proposed by Achenbach [6,7] and the action-thought theory [8], depressive symptoms could be delineated into internalizing and externalizing symptoms. Internalizing symptoms are those that are more thought or emotion oriented (depressed mood, feelings of worthlessness/hopelessness, feelings of guilt, suicidal ideations/attempts), whereas externalizing symptoms are behavioral and action oriented (irritability, aggressive behavior, changes in psychomotor patterns). Although aggressive behavior is not listed among the DSM-IV criteria, it is found in research reports on depression in young children [9]. The remaining symptoms can be seen as physiological symptoms of depression with no visible differences across developmental levels [2].

Chronological age (CA) is not completely indicative of developmental level; variability exists in how quickly children achieve cognitive and emotional skills, just as observed in physical development. Therefore, CA alone is not typically a precise measure of developmental level [10]. IQ also predicts children's rate of progression through development; but IQ is unrelated to developmental level, as it is normed to age. A better option would be to use both CA and IQ, which is known as mental age (MA) [5]. These are not, however, ideal to indicate true developmental level, which encapsulates physical, emotional and social development in addition to cognitive [11].

Only one empirical investigation [12] to date has directly tested the hypothesis of a relationship between developmental level and depressive symptom patterns. Participants of this study were psychiatric outpatients and were between the ages of 8 and 13 years of age. Using depression diagnoses based upon the DSM-III, children's depressive symptoms were identified through a structured interview. Developmental level was determined through pubertal and cognitive stages. The results of the study indicated that there was no relationship between the identified developmental level and children's pattern of depressive symptoms. One possible explanation for the negative results of this study could be the very restricted range of ages represented in the sample (45% of the children were 10 or 11 years old).

The purpose of the present study is to re-investigate the relationship between developmental level, using MA (IQ multiplied by CA and divided by 100) and symptom patterns in depressed children. It is first expected that, as the children's MA increases, so should the number of internalizing symptoms, operationalized as the ratio of internalizing symptoms to total symptoms identified. It is also anticipated that when MA is split at the median score, low and high MA should delineate between internalizing and externalizing symptoms. Additionally, because childhood depression often presents concurrently with anxiety and conduct disorders, MA may also be related to the presence of these diagnoses [13]. Therefore, it is hypothesized that children with lower MAs will have a higher rate of comorbidity with conduct disorders (more action based symptoms) than with only anxiety disorders (more thought based symptoms). Finally, the relationship between demographics (gender and ethnicity) and depressive symptomology will be investigated. In terms of gender, multiple studies have indicated that males present with predominantly externalizing symptoms and females, internalizing [14]. Some research has suggested, however, that maturation level largely accounts for gender differences on many variables, including psychiatric symptoms [15]. It is therefore predicted that females will have higher internalizing symptoms and will be at a higher developmental level. As culture and ethnicity have also been shown to have an effect on psychopathological symptoms [16], exploratory analyses will be conducted to evaluate the relationship between ethnicity and depressive symptoms.

## Methods

### Participants

Participants were 252 current or past patients from a children's psychiatric inpatient service within an urban hospital setting from 2000 to 2005. Although inpatient children are not representative of all children due to the severity of their symptoms and subsequent functioning difficulties, they were utilized in the present research study because they were fully experiencing severe depression. Additionally, a large quantity of detailed records (including symptom notes from staff and clinicians, parent or guardian reports, observable child behavior and survey scores) can be used within this population. Given these reasons, using an inpatient sample provides a good degree of power to identify the relationship between developmental level and patterning of depressive symptoms, if indeed such a relationship exists.

The inpatient service is a 15-bed facility that provides comprehensive psychiatric, psychosocial and educational evaluation for children aged 4 to 16. Children are typically referred from the emergency room at the general hospital or other local hospitals. Children were only included in the study if they had been given an IQ test and were diagnosed with a depressive disorder; there were no other exclusion criteria. Out of a total number of 716 individual children who were admitted to the psychiatric hospital between 2000 and 2005, 350 (48%) received depressive diagnoses. Of these 350, 252 (72%) had IQ test results in their records and were therefore included in this study. The children missing IQ results were not significantly different in Title 19 (governmental medical assistance which is indicative of poverty level) or Department of Child and Families status (DCF; child welfare services) from children who had IQ tests, *p *> .05.

Patients were predominantly male (60.7%) and their ethnicities were varied: Caucasian (54.4%), African American (21.8%), Hispanic (19%), Multi-racial (4.4%) and Asian (0.4%). Forty-seven percent of the sample qualified for Title 19 services and 17% were affiliated with DCF. Participants' ages ranged from 4 to 16 years old, with an average of 10.23 (*SD *= 2.42) years. Participants' mental ages were more varied, ranging from 3 to 19, with an average of 9.78 (*SD *= 3.07) years. The majority of participants were diagnosed with Depressive Disorder NOS (38.1%) or Mood Disorder NOS (31.7%), followed by Major Depressive Disorder with psychotic features (12.7%), Major Depressive Disorder (11.5%), Major Depressive Episode (3.2%) and Dysthymia (2.8%). The majority of participants (66%) were comorbid for an anxiety or conduct disorder (25% for an anxiety disorder, 29% for a conduct disorder, and 12% for both an anxiety disorder and a conduct disorder). All diagnoses were made by experienced clinicians using DSM-IV TR criteria. Children experienced a wide spectrum of depressive symptoms, with most symptoms being experienced by more than 60% of the sample. A few symptoms, such as feelings of guilt (8%) and diminished ability to concentrate (16%) were not very prevalent within this sample. The average IQ score of this sample was 92.65 (*SD *= 16.5) and was normally distributed. It is worthy to note that only 5% of the sample was diagnosed with mental retardation, indicating that the vast majority of the sample was cognitively normative.

### Measures

Depressive symptoms, using DSM-IV definitions, were established by reviewing and noting symptom presence on each patient's complete record, which included checklists of symptom presentation, daily notes and a discharge summary. Within each child's record, raters searched for the presence of seven symptoms which would follow Achenbach's conceptualization: depressed mood, worthlessness/hopelessness, guilt, suicidal thoughts/attempts, irritability, aggression and changes in psychomotor patterns. Symptoms were noted as either present or absent within the record; the child was given a score of 1.0 for every symptom present. Inter-rater reliability among the four raters involved in the project was established at 93% (average κ = . 86). Depressed mood, worthlessness and hopelessness, feelings of guilt and suicidal thoughts or attempts were combined into an internalizing symptom score, then divided by the number of total symptoms (of the seven symptoms studied) to create the internalizing ratio score. The rationale for using a ratio score is that it will take into account both internalizing and externalizing scores, as well as indicate the proportion of internalizing as hypothesized.

The majority of children (76%) were given a Kaufman Brief Intelligence Test I or II (K-BIT) by hospital staff during the time of their stay [17,18]. Occasionally, children had been given a Wechsler Intelligence Scale for Children (23%; WISC) or the Wechsler Preschool and Primary Scale of Intelligence (1%; WPPSI) by outside sources prior to their admission [19-21]. The K-BIT is a brief measure of verbal and nonverbal intelligence designed for children aged 4 years and older, which has both established reliability and validity, with internal consistency reliabilities averaging .94 for the overall K-BIT IQ Composite, .93 for the Vocabulary subtest, and .88 for the Matrices subtest [17,18]. The reliability and validity of Wechsler IQ tests have been well established, with the majority of subscales maintaining an internal consistency of at least .79, test-retest reliability of .76 or better, and validity correlations of .79 or higher [19-21]. In the present study, children's MA was calculated by multiplying a child's CA by their Full Scale IQ score and dividing it by 100. Even though MA is not a perfect measure of developmental level, it is a simple, brief and singular measure which has both clinical application and significance for the children within this sample.

### Procedure

IRB approval was obtained in order to review children's established records. Since this information was stored in an anonymous database and there were no risks or interventions, no consent was required. The list of all patients admitted to the psychiatric hospital since the year 2000 was reviewed to identify all children with depressive diagnoses. These participants were then screened for IQ scores and the child's discharge report for the first admission to the hospital was identified and reviewed. All demographic and diagnostic data were noted through the record review or by searching the hospital patient record computer database.

## Results

**Figure 1:**
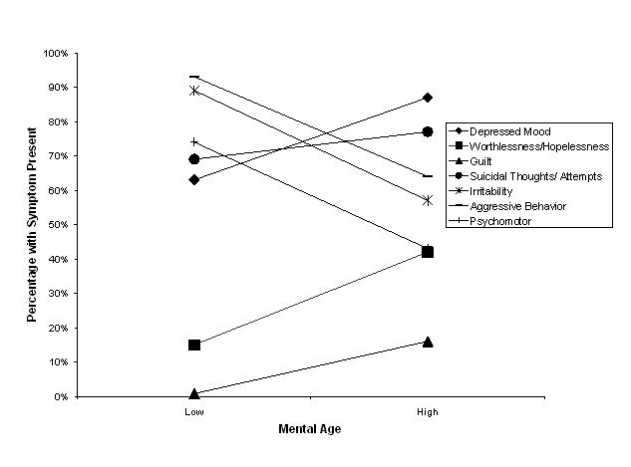
Internalizing and externalizing symptoms by developmental level.

The first hypothesis, that mental age would be positively correlated with internalizing depressive symptoms was tested with a more stringent alpha level of <.01 in order to account for inflated error. A Pearson's correlation was used to identify whether or not a relationship existed between MA and the internalizing ratio score. The sample's mean internalizing ratio score was .49 with a standard deviation of .25. The results indicate that mental age was a strong correlate of internalizing depressive symptoms, *r *= .51, *p *< .0001. It is interesting to note that the correlation was slightly larger than that between chronological age and the internalizing ratio score, *r *= .48, *p *= .0001, and much larger than that between IQ and the ratio score, *r *= .249, *p *= .0001. For the purpose of investigating the patterns of individual internalizing and externalizing symptoms, MA was split at the median (*Mdn *= 9.47) into a low and high category. An independent t-test revealed several significant findings, as indicated in Table 1. The pattern of individual symptoms across developmental level can be seen in Figure 1. Whereas aggressive behavior, irritability, and psychomotor changes decrease across the two developmental time periods, depressed mood, worthlessness and hopelessness and guilt increase.

**Table 1:** Descriptive Statistics Of and Significance Tests Between Participants Presenting with Symptoms by Low (n = 126) and High MA (n = 126)

Depressive Symptom	Low MA			High MA			t	*df*	*p*
	n-sym	*M*	*SD*	n-sym	*M*	*SD*			
Depressed Mood	78	.63	.49	111	.87	.33	4.69*	222	<.0001
Worthlessness and Hopelessness	18	.14	.35	54	.43	.50	5.27*	226	<.0001
Feelings of Guilt	1	.01	.16	20	.16	.37	4.48*	140	<.0001
Suicidal Thoughts or Attempts	86	.68	.47	98	.78	.42	1.71*	247	.09

Irritability	110	.89	.32	74	.57	.50	6.05*	212	<.0001
Aggressive Behavior	115	.93	.26	82	.64	.48	6.01*	191	<.0001
Changes in Psychomotor Patterns	92	.75	.44	55	.42	.50	5.52*	246	<.0001

Note: n-sym indicates the number of participants who had that particular symptom present (a score of 1).

* indicates that t-test score was adjusted for equal variances not assumed, as determined by a Levene's test for Equality of Variances, p < .01.

The depressive symptom analysis did indicate that one internalizing symptom did not change with the developmental level groupings: suicidal thoughts and/or attempts. In terms of suicidal ideations and attempts, the presence of suicidality was observed from a mental age of 4 years. However, two interesting qualitative details were noted throughout the record reviews. The first was that the suicidal methods children proposed or acted upon were very different according to age. Numerous younger children voiced "I want to throw myself in front of a car," "jump out of a window," or "jump off of a roof." Older children seemed more likely to plan or take action to take an overdose of pills, cut their wrists, hang or shoot themselves. Second, numerous children, especially those at young ages, presented with serious self-injurious behavior (SIB) such as stabbing themselves with a pen or breaking glass and cutting themselves with it. This type of SIB seemed to have a suicidal intent although frequently it was not voiced directly by the children involved.

**Figure 2:**
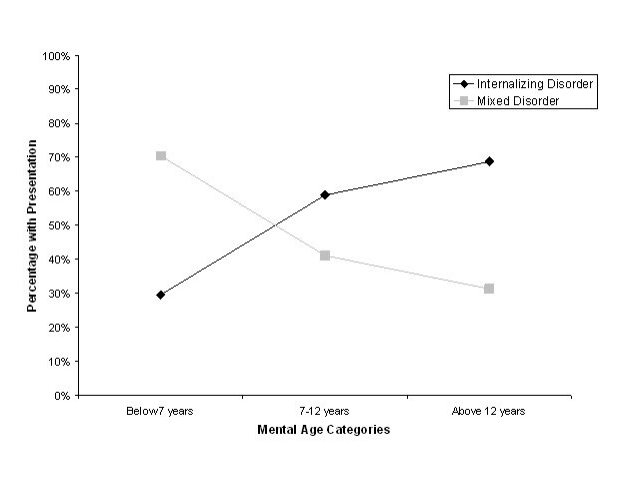
Internalizing and mixed presentation of disorders by developmental level.

The second hypothesis was that mental age would be related to comorbid disorders. In order to investigate this hypothesis, percentages of children with a depressive disorder diagnosis only or a comorbid anxiety disorder were collapsed into an 'internalizing only' category to indicate that the children presented with only internalizing disorders. Those with a comorbid conduct disorder present were placed into a 'mixed disorder' presentation using age categories supported by past research. Children were separated into 3 categories: below 7 (n = 27; 29.6% internalizing; 70.4% mixed), 7 to 12 (n = 158; 58.9% internalizing, 41.1% mixed) and above 12 mental age (n = 67; 68.7% internalizing, 31.3% mixed). As shown in Figure 2, internalizing disorders increased linearly with mental age categories, while the presentation of mixed disorders decreased. A Chi-Square test revealed that these values were significantly different from chance, *X*^2^*(2,N = 252) *= 12.11, *p *= .002.

To evaluate the role of gender, a factorial ANOVA with gender and the low and high mental age categories were used as independent variables; the internalizing ratio score was entered as the dependent variable. Within the low MA group, males were lower internalizers (*M *= .36, *SD *= .20) than their female counterparts (*M *= .41, *SD *= .18). This difference between males (*M *= .55, *SD *= .25) and females (*M *= .66, *SD *= .26) stayed consistent in the high MA group. There was a main effect for gender, *F(1,248) *= 7.81, *p *= .006, and mental age, *F(1,248) *= 52.69, *p *< .0001, but there was no interaction between the variables, *F(1,248) *= 1.22, *p *= .27. In order to identify if gender would still contribute significantly to depressive symptoms after mental age was controlled, a hierarchical regression analysis was computed. The results indicate that even when MA is controlled for, gender still contributes a significant amount of variance to the internalizing ratio score, *B *= .09 *(SE = .03)*, p < .01.

**Figure 3:**
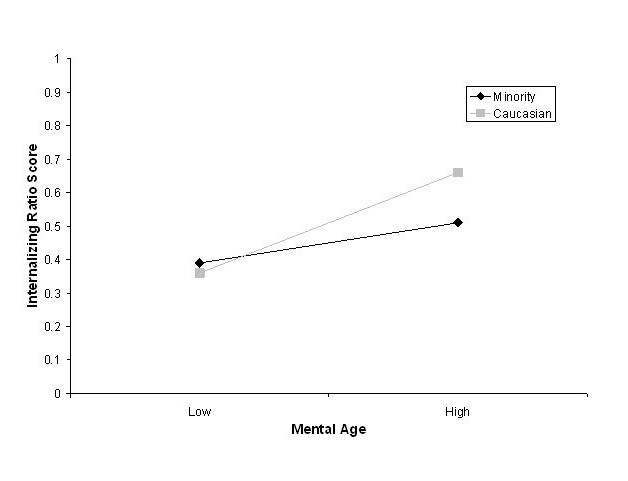
Interaction between Mental Age and Minority Status on internalizing ratio score.

Before running the full analysis on ethnicity, the differences between the individual minority categories (African American, Hispanic and Multi-racial) on the internalizing ratio score were evaluated to identify if collapsing the categories into a minority variable would be appropriate. There were no differences between the ethnicities on the internalizing ratio measure, *F(2,113) *= 0.08, *p *= .93; therefore, the ethnicities were transformed into a minority variable. Similar to the analysis for gender, a factorial ANOVA with minority status (minority versus Caucasian) and the low and high mental age categories were used as independent variables and the internalizing ratio score was entered as the dependent variable. However, because race and poverty level are often confounded, in this variable Title 19 status was controlled by using it as a covariate. Minorities (*M *= .39, *SD *= .20) were on par with Caucasian participants (*M *= .36, *SD *= .19) within the low MA category, but at the high MA levels, minority children (*M *= .51, *SD *= .21) were lower in internalizing symptoms than the Caucasian children (*M *= .66, *SD *= .26). Using Title 19 as a covariate, there was still a main effect for mental age, *F(1,247) *= 49.63, *p *< .0001, but no main effect for ethnicity, *F(1,247) *= 4.53, *p *= .04. There was a significant interaction between the variables, *F(1,247) *= 8.96, *p *= .003 (See Figure 3).

## Discussion

The hypothesis that mental age would be associated with symptom presentation of depression was supported. Mental age served as a correlate of depressive symptoms and the relationship can be seen across developmental time periods. Mental age was found to be a much better predictor than IQ; however, in this predominantly developmentally-normative sample, CA was also a good predictor, indicating that CA has value as a predictor of symptoms as well. These results are supported by previous research and theory on depression across different ages and IQs, as well as normative developmental theory [22]. The findings from this study differ from the results of the Kovacs and Paulauskas [12] study, which did not find a significant relationship between developmental level and depressive symptoms, which may be because this study had participants with a larger age range.

The failure of suicidality to differ between the developmental time periods may be due to the failure to distinguish between gestures, serious intentional SIB and attempts. SIB is noted to occur in children as young as 3 years old [2,23]. Suicidality is not a singular concept: it may be divided into serious SIB, suicidal thoughts and actual suicidal attempts. Since the present study did not separate out thoughts and attempts and did not include SIB, it is impossible to say how development is realistically related to suicide in this sample. It may be that very young children present with more immature suicidal intent (such as serious SIB), and as development progresses, suicidal thoughts and attempts begin to emerge.

The hypothesis that mental age would correspond to the type of comorbid diagnoses was supported. Comorbidity was related almost linearly with mental age and presentation (internalized only or mixed presentation), which supports the numerous studies reporting the emergence of conduct disorders before anxiety disorders [4]. This relationship could be explained in that anxiety disorders may be less prevalent in very young children because certain cognitive structures need to have developed to express anxiety in a traditional manner. Conduct Disorders, on the contrary, may be completely action-based in nature and may not require the development of certain cognitive functions. Another reason for this finding could be that depression and these other disorders are not comorbid at all; rather they are extended symptoms of depression. When a child presents with depressive symptoms and particular conduct symptoms, do they really only have depression or do they have a comorbid conduct disorder? The answer to this question remains elusive and the issue itself continues to be an area of contention in the field [24].

Gender, even when controlling for MA, had a significant relationship with depressive symptomology, which is consistent with previous research that indicates a possible socialization or biological difference for these behaviors [25,26]. However, if a more complete measure of developmental level (which includes social development) was used, then developmental level might have accounted for the differences found between the genders.

Although there was no main effect for ethnicity, there was a significant interaction between ethnicity and mental age. At a low developmental level, there was no significant difference between minorities and Caucasians on the internalizing ratio score – they were both quite low. However, in the high mental age group, minorities were significantly lower on the internalizing ratio score than their Caucasian counterparts. This indicates that the relationship between developmental level and the internalizing ratio score is buffered by a cultural variable. Familial and cultural socialization has been shown to promote aggression and eschew suicide in minority populations [27]. Perhaps then, this socialization, although individually not stronger than mental age, dampens the relationship between developmental level and symptoms in higher mental age periods, such as adolescence.

## Conclusion

The findings presented within this study indicate that a developmental approach is useful in understanding children's depressive symptoms. Within the context of gender and culture, children's symptom presentation was significantly related to their age. This indicates that as a child develops, their experience of depression changes in important ways. These differences can complicate both the diagnosis and treatment of depression in children. By increasing the knowledge of how depressive symptoms change across the course of childhood, earlier diagnoses of depression in children can be made and the best treatment options can be selected.

This study has several limitations. First, the findings of this study may not be generalizable to all children due to the inpatient sample used. These children did vary ethnically, but nearly half of the sample was below poverty level and 20% of these children had been removed from their homes by the Department of Children and Families. The level of aggression and suicidal behavior in this sample was quite high, as this was often the primary reason for admittance to the hospital. Therefore, these findings must be accepted with caution until they are replicated and validated. Second, this study was limited by the completeness and accuracy of the hospital records. Third, as mentioned earlier, MA is an incomplete proxy for developmental level, which could have limited the power of the study. Finally, all analyses were completely correlational and, as such, all results indicate purely the presence of a relationship.

Subsequent research should focus on replication of this study, using both MA and a broader measure of developmental level, either in an outpatient or community sample. It would also be interesting to identify if these findings generalize to other psychiatric disorders. It is probable that the relationship between developmental level and symptomology could be universal and extend past depressive disorders. Another direction for future research would be to investigate why some internalizing symptoms (depressed mood and suicidality) occur more often in children, whereas feelings of guilt and worthlessness/hopelessness are less frequent, even in adolescence. Equally important would be to investigate the role and interaction of socialization (as purportedly noted in gender and ethnicity) with developmental level and depressive symptoms.

Although the data presented in this study quantitatively describes the experience of depression in children, it can not fully convey the extent of the distress that depression can cause to the child and their family. Many of the children with lower MAs presented with such extreme behavioral problems that depression was only identified within the inpatient facility by the trained clinicians. Some depressed children's behaviors included urinating defiantly on a sibling's bed, severe SIB (e.g., stabbing self with a pen) and persistent aggressiveness towards animals, friends and parents or guardians. After several weeks in an inpatient setting, clinicians uncovered events which would understandably cause feelings of depression, such as being abandoned, being excessively bullied at school and having parents who are currently in the process of a divorce. These children were then diagnosed as having a depressive disorder and given the appropriate treatment. One must question the possibility that if aggression and other externalizing behaviors were well known as symptoms of depression for young children, early identification could have been made. The data presented here suggests that knowing a child's developmental level is important for early and accurate diagnosis and treatment decisions.

## Abbreviations

ANOVA- Analysis of Variance

CA- Chronological Age

DCF- Department of Children and Families

DSM-IV - Diagnostic and Statistical Manual of Mental Disorders

KBIT- Kaufman Brief Intelligence Test

MA- Mental Age

NOS- Not Otherwise Specified

WISC- Wechsler Intelligence Scale for Children

WPPSI- Wechsler Preschool and Primary Scale of Intelligence
